# Application of Fucoidan in Caco-2 Model Establishment

**DOI:** 10.3390/ph15040418

**Published:** 2022-03-30

**Authors:** Qiong Yang, Maochen Xing, Ke Wang, Qiang Wei, Jiarui Zhao, Yuan Wang, Kai Ji, Shuliang Song

**Affiliations:** 1 Marine College, Shandong University, Weihai 264209, China; yangqiong1237@163.com (Q.Y.); sddxxmc@163.com (M.X.); wk1308866506@163.com (K.W.); wq18086420598@163.com (Q.W.); 201936684@mail.sdu.edu.cn (J.Z.); wangyuan1126@mail.sdu.edu.cn (Y.W.); 2 Department of Plastic Surgery, China-Japan Friendship Hospital, Beijing 100029, China; 3 Shandong University-Weihai Research Institute of Industrial Technology, Weihai 264209, China

**Keywords:** fucoidan, Caco-2 model, absorption of macromolecules

## Abstract

The Caco-2 model is a common cell model for material intestinal absorption in vitro, which usually takes 21 days to establish. Although some studies have shown that adding puromycin (PM) can shorten the model establishment period to 7 days, this still requires a long modeling time. Therefore, exploring a shorter modeling method can reduce the experimental costs and promote the development and application of the model. Fucoidan is an acidic polysaccharide with various biological activities. Our study showed that the transepithelial electrical resistance (TEER) value could reach 600 Ω·cm^2^ on the fourth day after the addition of fucoidan and puromycin, which met the applicable standards of the model (>500 Ω). Moreover, the alkaline phosphatase (AKP) activity, fluorescein sodium transmittance, and cell morphology of this model all met the requirements of model establishment. Fucoidan did not affect the absorption of macromolecular proteins and drugs. The results indicate that fucoidan can be applied to establish the Caco-2 model and can shorten the model establishment period to 5 days.

## 1. Introduction

The Caco-2 cell line is a human clonal colonic adenocarcinoma cell. Its structure and physiological functions are similar to those of human intestinal epithelial cells, and they contain enzymes related to intestinal epithelial cells. Caco-2 cells can spontaneously undergo epithelioid differentiation under in vitro culture conditions, forming the same microvilli structure [1] and tight junctions as intestinal epithelial cells. As a result of the similarity to the intestinal columnar epithelial cells in the morphology, functional expression of marker enzymes, and the permeability, Caco-2 cells are often used to study the transport [2,3,4], absorption, and permeability [5,6] of substances in the intestine and the effects of pharmaceutical dosage forms, prodrugs, carriers, and structures on absorption.

Substances studied using this model include natural products and compounds, such as ideain, amphotericin B, tubulin inhibitor YMR-65, and natural compound Datoraolone isolated from Datura Innoxia Mill [7,8,9,10]. Caco-2 cells with a specific density were seeded on a polycarbonate fiber membrane, after 21 days of culture, causing the surface of Caco-2 cells to show dense microvilli; this state of differentiation enables Caco-2 cells to have similar material transport conditions [11,12], and metabolic enzymes [13] needed to study drug absorption and transportation as normal intestinal epithelial cells, such as studying the transport of drugs via the paracellular pathway, transcellular diffusion, or studying the mechanisms of active uptake and efflux of drugs [14,15,16]. The traditional Caco-2 model requires at least a 21-day-long culture period to fully differentiate the monolayer of Caco-2 cells, which limits the yield and practicability of the model to a certain extent. 

Therefore, faster and more efficient cell culture processes will provide a more cost-effective process for screening compounds [17]. To adapt the model to the needs of rapid screening, many efforts have been made by researchers to speed up the preparation of Caco-2 cell monolayers. For example, cell culture using media containing a combination of calf serum supplemented with 2% iron, growth factors, and hormones [18] usually reduces the modeling period of the Caco-2 model by 3 days. 

Recent studies have indicated that adding Puromycin to the Caco-2 model can reduce the modeling period to 7 days [17], possibly because the addition of puromycin can cause Caco-2 cells to differentiate and can enhance cell barrier properties. Moreover, this process can increase the p-glycoprotein (P-GP) transcriptional expression level [17,19,20]. Although this finding significantly reduced the modeling time, it still required a long modeling time, and the experimental costs and risk of experimental infection were high. Therefore, exploring a shorter modeling method can reduce the experimental costs and increase the benefits as well as promote the development and application of the model.

**Figure 1:**
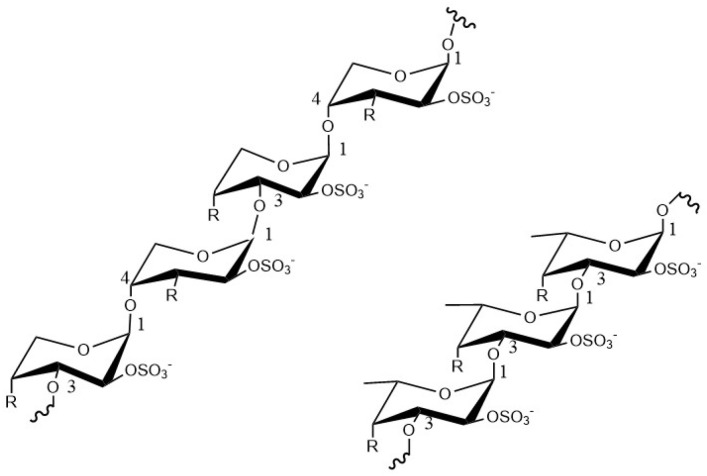
The structure of fucoidan.

Fucoidan is a complex water-soluble sulfated polysaccharide, which is usually derived from the cell wall of brown algae and some marine invertebrates [11,21] and is typically composed of fucoidan polymers linked by α (1→3) and α (1→4) and some sulfate groups show in Figure 1. Fucoidan also contains a proportion of other monosaccharide compositions, including alduronic acid, galactose, xylose, mannose, rhamnose, glucose, and arabinose [12,13]. Fucoidan is a natural product, and many studies have shown that fucoidan has low toxicity and side effects, and generally does not cause adverse reactions in the body [22,23,24]. 

In our laboratory’s previous studies on the absorption mechanism of fucoidan, we found that fucoidan can promote the increase of transepithelial electrical resistance (TEER) value of Caco-2 cells, and by reviewing the relevant literature, we found that fucoidan can play a protective role in the intestinal barrier by regulating tight junction protein expression [25,26]. Therefore, we suggested that fucoidan may promote the establishment of the Caco-2 model. However, a further review of the literature revealed no research on whether fucoidan could shorten the period for the Caco-2model establishment and whether it could be applied to the Caco-2 model.

In this study, we examine the effect of fucoidan on Caco-2 model establishment by adding it during the modeling process. First, the TEER value, AKP enzyme activity, electron microscope morphology, and fluorescein sodium permeability are used to verify whether the Caco-2 model established under the influence of fucoidan reached the standard of model establishment. The differences between the 5-day Caco-2 model and the 7-day Caco-2 model in both macromolecule protein and macromolecule drug absorption were detected, further verifying the success of the 5-day Caco-2 model. 

## 2. Results

### 2.1. Fucoidan Had no Significant Effect on the Activity of Caco-2 Cells

Before the experiment, we first tested the effect of fucoidan on the proliferation activity of Caco-2 cells. Fucoidan has low toxicity and side effects, and research showed that it has no inhibitory effect on Caco-2 cells when the concentration of fucoidan is 1 mg/mL [27]. In addition, our laboratory previously studied the absorption and distribution of fucoidan and found that fucoidan can promote the resistance value of the 7-day Caco-2 model within the concentration range of 20–200 µg/mL [28]. 

**Figure 2:**
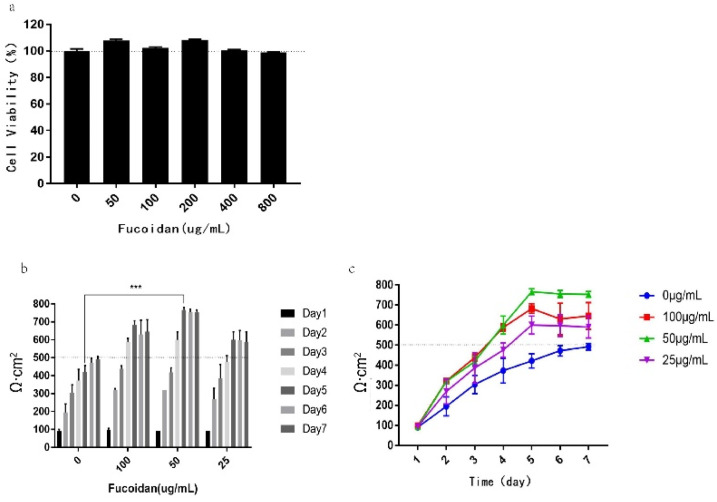
Effect of fucoidan concentration on Caco-2 model establishment. (**a**) The cell viability in macrophages detected after incubation with fucoidan for 24 h by MTT (3-(4,5-dimethylthiazol-2yl)-3,5-diphenytetrazolium bromide). (**b**) Histogram of TEER value growth under different fucoidan concentrations. (**c**) Line chart of TEER value growth trend at different fucoidan concentrations. The data is shown as ± SD (*n* = 3); *** *p* < 0.001.

Therefore, we detected its effect on the activity of Caco-2 cells in the concentration range of 0–800 µg/mL. The results are shown in Figure 2a; when the fucoidan concentration is 50, 100, 200, 400, and 800 µg/mL, the cell viability did not decrease compared with the control group, and the cell proliferation was promoted in the range of 50–100 µg/mL. The results indicate that fucoidan had no toxicity to Caco-2 cells within the range of tested concentration and that it could be used for subsequent experiments.

### 2.2. Fucoidan Can Shorten the Modeling Period to 5 Days and the Best Effect Is When the Concentration Is 50 µg/mL

To study the effect of fucoidan concentration on the Caco-2 model, Caco-2 cells were treated with different concentrations of fucoidan, and the changes of the transepithelial electrical resistance (TEER) in transwell cells were observed and recorded. The results are shown in Figure 2b,c; the modeling resistance value of the fast 7-day Caco-2 model established by E. Sevinet al. reached 492 Ω·cm^2^ on the seventh day. However, adding 25–100 μg/mL fucoidan can significantly accelerate the increase in the TEER value during this process, making the resistance value reach 500 Ω·cm^2^ on the fifth day and keeping the resistance value > 500 Ω·cm^2^ the entire time.

In combination with the above experimental results, it is shown that adding 25–100 µg/mL fucoidan in the Caco-2 cell modeling process can promote the establishment of the Caco-2 model. However, at a concentration of 100 µg/mL fucoidan, the resistance value decreased slightly on the sixth and seventh days, and it is difficult to maintain at a stable level. Although the resistance value under the concentration of 25 µg/mL fucoidan can reach 500 Ω·cm^2^ on the fifth day, it promotes the increase of resistance value slowly, and the maximum can only reach 600 Ω·cm^2^. 

At the concentration of 50 µg/mL fucoidan, the resistance rate increased the fastest, reaching 600 Ω·cm^2^ on the fourth day, and reaching the maximum value of 776 Ω·cm^2^ on the fifth day, which was maintained at a relatively stable level afterward. Therefore, the concentration of 50 µg/mL fucoidan was selected as the concentration of fucoidan used in subsequent modeling.

Although the addition of fucoidan can make the TEER value reach the model establishment standard on the fifth day, whether the established 5-day Caco-2 model can be used in the experiment requires further verification.

### 2.3. The AKP Enzyme Is Secreted Normally and the Function of Caco-2 Cells Is Not Affected in the 5-Day Caco-2 Model

To verify the accuracy of the 5-day Caco-2 model, the AKP activity in the AP and BL chambers (AP/BL) of the Caco-2 model was investigated on the fifth day. During the establishment of the Caco-2 model, Caco-2 cells undergo epithelial-like differentiation and gradually form a polarized monolayer with a top brush edge on the intestinal lumen side. AKP is a marker enzyme of the intestinal epithelial brush border cells. Its concentration represents the polarization and function of the Caco-2 monolayer [29,30].

**Figure 3:**
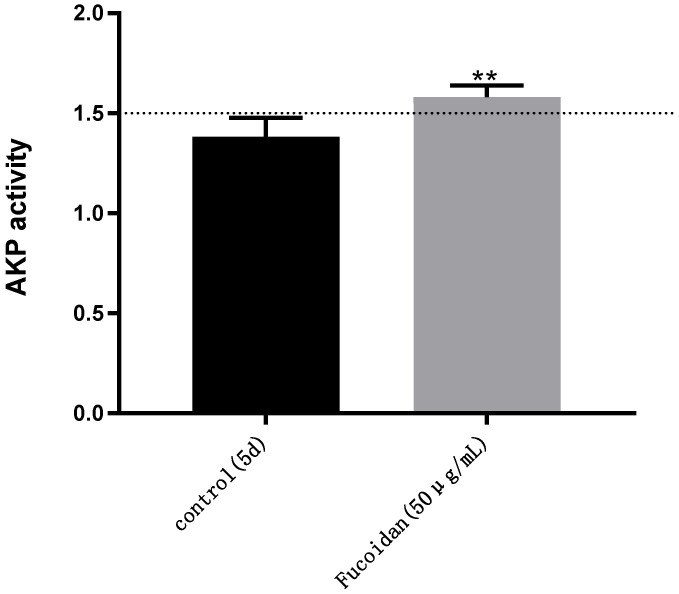
AKP activity: detection of AKP activity in different Caco-2 models on the fifth day (AP/BL). Control: DMEM containing 0.4 µg/ mL puromycin. Fucoidan: DMEM containing 0.4 µg/mL puromycin and 50 µg/mL fucoidan. The two models were cultured at the same time and under the same conditions. Significance test: a *t*-test was conducted; ** *p* < 0.01.

The results are shown in Figure 3; on the fifth day, the AKP (AP/BL) activity of the Caco-2 model (control) without fucoidan was 1.38, which did not meet the modeling standard (AKP > 1.5). The AKP activity (AP/BL) of the Caco-2 model established under the influence of 50 μg/mL fucoidan was 1.58—higher than 1.5 [31]. Thus, the 5-day Caco-2 model established by adding 50 µg/mL fucoidan showed a seriously uneven distribution of AKP enzymes and obvious polarization of Caco-2 cells, which met the standard of the Caco-2 model.

### 2.4. Caco-2 Cell Morphology and Function Were Not Affected in the 5-Day Caco-2 Model

The small intestine is the primary site of digestion and absorption as well as the main organ of absorption. Small intestinal villi are microvilli structures existing on the surface of the small intestinal epithelial cells, which enlarges the cell surface area and facilitate the absorption of substances, and are related to cell metabolism. 

**Figure 4:**
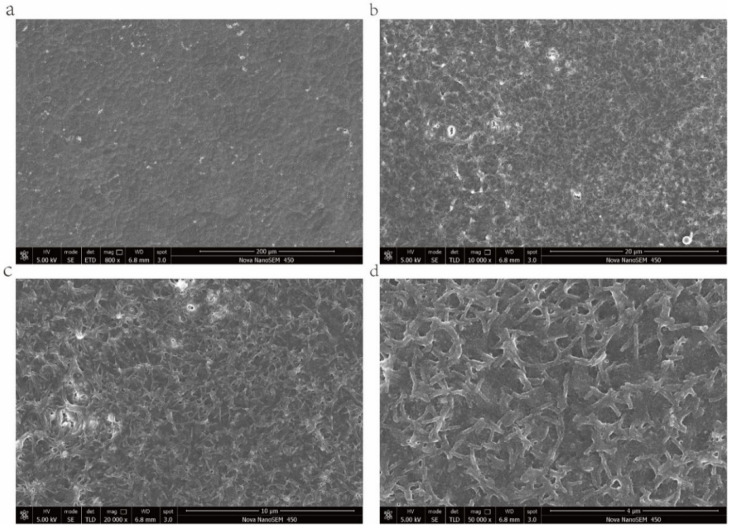
Morphology of monolayer Caco-2 cells under scanning electron microscope. (**a**) Caco-2 cell monolayer, Bar = 200 μm; (**b**) Caco-2 cell monolayer, Bar = 20 μm; (**c**) Caco-2 cell monolayer, Bar = 10 μm; and (**d**) microvilli structure, Bar = 4 μm.

As shown in Figure 4; at low magnification, Caco-2 cells were closely arranged, each cell was irregularly round, and the boundary between cells was clearly visible (Figure 4). At 10,000 and 20,000 times magnifications, irregular microvilli can be seen on the cell surface, and large cracks can be seen on the cell layer, which may be due to damage induced by the freeze-drying process to the cell monolayer model (Figure 4b,c). When magnified to 50,000 times, clear morphology of microvilli could be seen on the cell surface. The length and diameter of microvilli were approximately 600–800 and 80 nm, respectively, (Figure 4d), which agreed with previous studies [32]. The results indicated that, in the 5-day Caco-2 model, the Caco-2 cells grew and differentiated normally, the monolayer integrity of Caco-2 cells was good, and the morphological function of Caco-2 cells was not affected.

### 2.5. Fucoidan Did Not Affect the Permeability of the Caco-2 Model

Fluorescein sodium is an organic compound with a strong yellow-green fluorescence that has difficulty in penetrating the cell membrane; therefore, it can be used as a permeation marker [33] to detect the permeability of biofilms and can be detected by a fluorescence detector. The permeability of the 5-day Caco-2 model established by adding fucoidan was evaluated by measuring the difference in the concentration of fluorescein sodium between AP and BL transwell chambers.

The results showed that the apparent permeability coefficients of 25, 50, and 100 µg/mL fucoidan groups were all less than 1 × 10^−6^ within 30–180 min (Table 1), which reached the standard of the Caco-2 model [31] and had no significant difference compared with the 7-day Caco-2 model group. Among them, the Papp and RT (Table 1 and Table 2) of the 100 µg/mL fucoidan group were both lower than those of the 50 µg/mL fucoidan group, indicating that the permeability of the 100 µg/ mL fucoidan group was lower than that of the 50 µg/mL fucoidan group, which may be caused by the higher cell tight binding effect of 100 µg/mL fucoidan. However, compared with the previous experimental results (MTT and TEER in Figure 2), the effect was best when the concentration was 50 µg/mL. Based on the above results, we proved that the Caco-2 model was successfully established, and can be used to simulate in vitro drug intestinal transport experiments.

### 2.6. The Addition of Fucoidan Has No Effect on Transferrin Transport and the Absorption of Materials Mediated by Clathrin and Caveolin

The Caco-2 model is usually used to simulate drug absorption, transport, and metabolism in the intestinal mucosa. To further verify the effect of the 5-day Caco-2 model with fucoidan on substance absorption, the differences regarding transferrin absorption between the 5-day and 7-day Caco-2 model established by E. Sevinet al. was investigated. Transferrin is the main ferric protein in plasma and can reversibly bind Fe^3+^ to form a complex in cells [34]. Simultaneously, transferrin is often used as a marker of caveolin and clathrin-mediated endocytosis [35].

**Figure 5:**
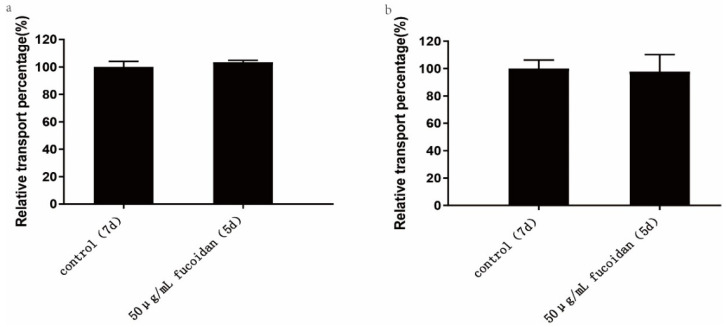
Effect of fucoidan on the absorption of macromolecules. The control group was the 7-day model, and the 50 µg/mL Fucoidan group was the 5-day Caco-2 model group established by us. The two models were cultured at the same time and under the same conditions, and the absorption of macromolecules was studied on the seventh and fifth day respectively. (**a**) Effect of fucoidan on FITC-transferrin absorption. (**b**) Effect of fucoidan on the absorption of FITC-dextran. The data is shown as ± SD (*n* = 3). Significance test: a *t*-test was conducted.

As shown in Table 3 and Figure 5a, the apparent permeability coefficients of FITC-transferrin absorption within 120 min in the 7-day and 5-day Caco-2 model were 4.45 ± 0.18 × 10^−5^ cm/s and 4.61 ± 0.05 × 10^−5^ cm/s, respectively. The results revealed that there was no significant difference in the absorption and transport of FITC-transferrin between the two Caco-2 models, and the addition of fucoidan did not affect caveolin and clathrin-mediated absorption.

### 2.7. The 5-Day Caco-2 Model Can Be Used to Study the Absorption of Macromolecular Substances, and It Does Not Affect the Absorption of Substances Mediated by the Macropinocytosis Pathway

Dextran is a homotype polysaccharide composed of glucose as a monosaccharide, which has a large molecular weight and is usually absorbed by the body via the macropinocytosis pathway [36]. By investigating the absorption differences of dextran between the two different models, we can evaluate whether the 5-day Caco-2 model can be applied to the absorption experiment of macromolecules and detect the effect of the addition of fucoidan on the macropinocytosis pathway.

As shown in Table 4 and Figure 5b, the apparent permeability coefficients of FITC-dextran absorption within 120 min for the 7-day and 5-day Caco-2 model were 4.56 ± 0.28 × 10^−6^ cm/s and 4.46 ± 0.57 × 10^−6^ cm/s, respectively. The results indicated that there was no significant difference in the absorption and transport of FITC-dextran between the two Caco-2 models. Therefore, adding 50 μg/mL fucoidan to the Caco-2 model had no significant effect on the absorption of macromolecules. The macropinocytosis pathway may not be affected by the addition of fucoidan.

## 3. Discussion

High-speed and efficient in vitro intestinal models can be used for drug discovery and drug activity detection [37] and are of great value to the pharmaceutical industry. At present, several types of in vitro models of the intestine have been created, including cell monolayers (mono- and co-cultures), multicellular three-dimensional (3-D), and tissue chip or microfluidic systems for the evaluation of intestinal drug permeability [38]. The Caco-2 model is one of the cell monolayers and is the most commonly used in vitro intestinal model. 

The Caco-2 cell line is derived from human rectal cancer and colon cells. It has a similar structure and biochemical effects to human intestinal epithelial cells and can differentiate into material transport conditions and required metabolic enzymes similar to normal intestinal epithelial cells when cultured in vitro [39]. Caco-2 cells can grow autonomously and spontaneously differentiate into intestinal epithelial cells when cultured on polycarbonate membranes, thereby, forming a continuous cell monolayer.

However, it usually takes 21 days to form a complete monolayer membrane. Although some researchers have shortened the model establishment time to 7 days by changing the medium or adding puromycin and other substances [16,17,40], there are still the problems of high cost, long time consumption, and easy bacterial contamination in the modeling process. Therefore, the search for a faster and more efficient cell culture process will also provide a more cost-effective approach for screening compounds.

The TEER value is significantly related to the degree of tight junctions between cells. The larger the value, the tighter the connection between cells [12,41]. Generally, when the resistance value is greater than 500 Ω·cm^2^, it means that the cells have formed a tight monolayer, which can be used for experiments [42]. Our experiment showed that adding 0.4 μg/mL puromycin and a certain concentration of fucoidan in the modeling process had a significant effect on the increase of resistance value, among which, 50 µg/mL fucoidan had the most obvious effect, reaching 600 Ω·cm^2^ on the fourth day and remaining at a relatively stable level thereafter. These results indicate that fucoidan can promote the monolayer establishment of Caco-2 cells and may shorten the modeling time to 5 days. However, whether the established 5-day Caco-2 model meets the modeling standards in terms of integrity, polarity, and permeability of a single cell layer requires further verification.

Therefore, we detected the content of AKP [13], an enzyme that signals the degree of single-cell layer polarization of Caco-2 cells, and the transmittance of fluorescein sodium [43], an indicator of single-cell permeability of Caco-2 cells. The results showed that, on the fifth day, AKP enzyme activity at the concentration of 50 µg/mL fucoidan met the Caco-2 model establishment standard (>1.5), while the group without fucoidan did not meet this standard and had a much lower concentration compared with the fucoidan group [31]. 

There was no significant difference in permeability between the 25–100 µg/mL fucoidan group and the 7-day model group, indicating that fucoidan in the concentration range of 25–100 µg/mL can promote the tight connection of cells, which can also be seen from the previous TEER value. In addition, according to the MTT value, TEER value, and Papp value, 50 µg/mL fucoidan had the best effect. The results of SEM also showed that 50 µg/mL Fucoidan could promote the polarization of Caco-2 cells, and the microvilli could be grown on the surface of Caco-2 cells on the fifth day. The results showed that the 5-day Caco-2 model could be used as a small intestinal drug absorption model.

Additionally, we also studied the difference in drug absorption between the 5-day and the 7-day Caco-2 model. The experimental results indicate that the two models had no significant differences in the absorption and transport of FITC-transferrin and FITC-dextran. The addition of fucoidan may not affect clathrin- and caveolin-mediated endocytosis and micropinocytosis-pathway-mediated substance absorption, which further proves the successful establishment of the 5-day Caco-2 cell model.

In conclusion, the 5-day Caco-2 model established in our experiment significantly reduced the modeling time (by at least 3/4) compared to the traditional 21-day Caco-2 model and was also two days shorter than the 7-day Caco-2 model. As the time of cell culture is significantly shortened, the risk of bacterial contamination in the process of culture is greatly reduced, which increases the operability of the experiment and greatly reduces the cost of the experiment. Therefore, it provides a faster and lower-cost method to build the traditional Caco-2 model. 

However, the mechanism of fucoidan promoting model establishment was not studied in this experiment. According to the literature reports, fucoidan can repair the damaged intestinal barrier, which may be because it can promote the expression of tight junction proteins, such as Claudin, Occludin, and ZO-1 [25,26], and thus we speculated that the mechanism of promoting the establishment of the Caco-2 model may be achieved by promoting cell tight junctions. In addition, in this experiment, we only detected the influence of the 5-day Caco-2 model on the absorption of macromolecules (such as dextran and transferrin) and did not study the applicability to the absorption of small molecules.

Therefore, we will further study the effect of this model on the absorption of small molecules in future experiments. At the same time, we will study the above proteins through transcriptomics and proteomics to explore and determine the specific action mechanism of fucoidan.

## 4. Materials and Methods

### 4.1. Drugs and Reagents

The fucoidan extracted from the brown alga Fucus vesiculosus was purchased from Sigma-Aldrich (St. Louis, MO, USA; F8190), containing fucose (33%), uronic acid (8%), sulfate (23%), and minor amounts of amino sugar and protein with a purity of 95% and a peak molecular weight of 675.6KDa as assessed using multi-angle laser light scattering (Sigma-Aldrich Customer/Technical Service). 

Fucoidan is dissolved in Dulbecco’s modified Eagle’s medium (DMEM, without double antibodies; Hyclone, Logan, Utah, USA), containing puromycin (Puromycin, PM, 0.4 μg/mL, Aladdin, Shanghai, China) and 15% fetal bovine serum (Biological Industries, Ra’anana, Israel) (PM-DMEM), stirred for 30 min at 25 °C, then filtered through a 0.22-µm pore size filter (Sartorius, Göttingen, Germany), and stored at 4 °C. Cell culture consumables were purchased from the Corning Corporation (Corning, New York, NY, USA). Other requirements include fetal bovine serum (Biological Industries, Ra’anana, Israel), streptomycin, penicillin, and trypsin (Solarbio, Beijing, China).

### 4.2. Caco-2 Cell Culture

The human colon adenocarcinoma Caco-2 cell line was purchased from the Kunming Cell Bank, Chinese Academy of Sciences (Kunming, China). Caco-2 cells were cultured in DMEM containing 15% (*V*/*V*) fetal bovine serum, penicillin, and streptomycin (100 U/mL). The cells were cultured in a 25-cm^2^ cassette flask and incubated in a CO_2_ incubator. The solution was then changed daily. When the degree of cell fusion was 80–90%, the cells were digested with 0.25% trypsin and 0.02% EDTA and passed in the ratio of 1:3. Additionally, 20–50 generations of cells (1 × 10^5^ cells/mL) were used in all experiments.

### 4.3. Effect of Fucoidan on the Activity of Caco-2 Cells

The cell viability was determined using 3-(4,5-dimethylthiazol-2yl)-3,5-diphenyl tetrazolium bromide (MTT) assay. The fucoidan solution was diluted with 15% DMEM to 50, 100, 200, 400, and 800 µg/mL. Caco-2 cells at the logarithmic growth stage were inoculated in 96-well plates and cultured in an incubator at 37 °C constantly for 24 h. After 24 h, the Caco-2 cells were changed to DMEM containing the above-mentioned different concentrations of fucoidan and further cultured for 24 h. Subsequently, 20 μL MTT (Sigma-Aldrich, St. Louis, MO, USA) solution was added to each chamber. After incubating for 4 h at 37 °C in the dark, the supernatant was discarded, and 100 µL DMSO (Sigma-Aldrich, St. Louis, MO, USA) was added. After 10 min of dissolving the insoluble formazan, the absorbance of each chamber was measured at 570 nm using 630 nm as a reference wavelength. The value of absorbance was used to determine the cell survival rate.

### 4.4. Effect of Fucoidan Concentration on the Establishment of Caco-2 Model

According to the method of E. Sevinet al. [17], Caco-2 cells were cultured in PM-DMEM containing 15% (*V*/*V*) fetal bovine serum. We added Caco-2 cells (8000 cells/well) to the Apical (AP) of transwell and added 1000 μL PM-DMEM to the Basolateral (BL), with three parallels in each group. After 12 h, the medium in the AP and BL of transwell was replaced with PM-DMEM containing different concentrations of fucoidan (Fuc-PM-DMEM, 100, 50, and 25 µg/mL), and cultured for 7 days with the fluid changed daily. During modeling, the TEER value of the Caco-2 cell monolayer was measured using a Millicell-ERS voltammeter at fixed time points daily to detect the effect of fucoidan on the increase of the monolayer trans-membrane resistance value of Caco-2 cells.

### 4.5. TEER Value Measurement

Cells in the logarithmic growth phase of 6-well plates were inoculated into a 0.4-µm transwell chamber, and the trans-membrane resistance value of the Caco-2 cell monolayer was measured at fixed time points daily with the Millicell-ERS voltammeter to detect the effect of fucoidan on the growth of monolayer trans-membrane resistance value of Caco-2 cell. We calculated the TEER value according to the following formula:

$$\mathrm{TEER}=({\mathrm{TEER}}_{T}-{\mathrm{TEER}}_{c})\times A$$

where TEER_T_ represents the TEER value (Ω) of the measured Transwell chamber with cells, TEER_C_ is the TEER value (Ω) of the transwell chamber in the control group, and A is the membrane area of the transwell cavity (0.336 cm^2^).

### 4.6. Effects of Fucoidan on AKP Activity

Approximately 20–50 generations of cells were resuscitated and cultured to the logarithmic growth stage. Caco-2 cells were added to the AP chamber of transwell with 50-μg/mL Fuc-PM-DMEM and PM-DMEM, respectively, at the rate of 8000 cells/well. We added 1000-μL corresponding media to the BL chamber for culture, and changed the solution daily. When the Caco-2 model established on the Fuc-PM-DMEM media reached the fifth day, the Caco-2 model established on the PM-DMEM reached the seventh day, and TEER > 500 Ω ·cm^2^, the AP and BL (AP/BL) AKP activity of the two models was detected according to the instructions of the alkaline phosphatase kit (Beyotime, Shanghai, China).

### 4.7. Effects of Fucoidan on the Morphology of Caco-2 Cells (Scanning Electron Microscopy [SEM])

We removed the 5-day Caco-2 model polyester fiber membrane with the cells, fixed the cells with 2.5% glutaraldehyde at 4°C overnight, and washed three times with 0.1-mol·L-1 PBS (pH = 7.2), 10 min/time. After 30%, 50%, 70%, 90%, and 100% alcohol gradient dehydration for 30 min each, the membrane was put in a −40 °C low-temperature refrigerator overnight. The samples were then lyophilized using a freeze dryer, the dried samples were fixed on the sample holder with conductive glue, sprayed with gold in a high vacuum film plating instrument, and then observed and photographed using a Nova SEMNANO450 field emission scanning electron microscope, and the acceleration voltage was 5 kV.

### 4.8. Effect of Fucoidan on the Permeability of Caco-2 Cells

Moreover, 20–50 generations of cells were resuscitated and cultured to the logarithmic growth stage. Caco-2 cells were added to the AP chamber transwell with 50-μg/mL Fuc-PM-DMEM and PM-DMEM, respectively, at the rate of 8000 cells/well. We added 1000 μL of corresponding media to the BL chamber for culture and changed the solution daily. When the Caco-2 model established on the Fuc-PM-DMEM reached the fifth day, the Caco-2 model established on the PM-DMEM reached the seventh day, and TEER > 500 Ω ·cm^2^, the Caco-2 cells were washed with HBSS buffer solution three times, then 400-μL HBSS buffer solution was added to the AP chamber, 1000 µL HBSS buffer was added to the BL chamber, and the cells were incubated in a CO_2_ incubator for 30 min for balancing. 

Then, HBSS buffer was drained from the AP and BL chambers, 250 µL 10 µg/mL fluorescein sodium (Solarbio, Beijing, China ) solution was added to the AP chamber, and 1000 µL HBSS buffer was added to the BL chamber, and the cells were cultured at 37 °C for 30, 60, 120, and 180 min, respectively. After incubation, 200 µL of the BL chamber transport medium was collected in all-black 96-well plates to determine the fluorescence sodium intensity and calculate the transport concentration and transmission rate of fluorescein sodium in both the 5-day Caco-2 and 7-day Caco-2 models, with three parallel sets for each group.

### 4.9. Effect of Fucoidan on Absorption and Transport of Macromolecular Proteins in Caco-2 Cells

Here, 20–50 generations of cells were resuscitated and cultured to the logarithmic growth stage. Caco-2 cells were added to the AP chamber of transwell with 50 µg/mL Fuc-PM-DMEM and PM-DMEM, respectively, at the rate of 8000 cells/well. We added 1000 µL of corresponding media to the BL chamber for culture and changed the solution daily. 

When the Caco-2 model established on the Fuc-PM-DMEM reached the fifth day, the Caco-2 model established on the PM-DMEM reached the seventh day, and TEER > 500 Ω ·cm^2^, Caco-2 cells were washed with HBSS buffer solution three times, and then 400 µL HBSS buffer solution was added to AP chamber and 1000 μL HBSS buffer was added to the BL chamber, and then incubated in a CO_2_ incubator for 30 min for balancing. Later, HBSS buffer was drained from both the AP and BL chambers, 250 µL 10 µg/mL FITC-transferrin (Jackson, Bar Harbor, Maine (ME), USA) solution was added to the AP chamber, and 1000 µL HBSS buffer was added to the BL chamber, and the cells were cultured at 37 °C for 120 min. After incubation, 200 µL BL chamber transport media was collected in all-black 96-well plates for fluorescence intensity measurement and the related apparent permeability coefficient calculation. Three parallel plates were set for each group.

### 4.10. Effect of Fucoidan on Macromolecule Drug Absorption in Caco-2 Cells

A total of 20–50 generations of cells were resuscitated and cultured to the logarithmic growth stage. Caco-2 cells were added to the AP chamber of transwell with 50 µg/mL Fuc-PM-DMEM and PM-DMEM, respectively, at the rate of 8000 cells/well. We added 1000 µL corresponding media to the BL chamber for culture and changed the solution daily. When the Caco-2 model established on the Fuc-PM-DMEM reached the fifth day, the Caco-2 model established on the PM-DMEM reached the seventh day, and TEER > 500 Ω ·cm^2^, Caco-2 cells were washed with HBSS buffer solution three times, then 400 µL HBSS buffer solution was added to the AP chamber, and 1000 µL HBSS buffer was added to the BL chamber, and then incubated in a CO_2_ incubator for 30 min for balancing. 

Subsequently, HBSS buffer was drained from both AP and BL chambers, 250 µL 10 µg/mL FITC-dextran (10kD, TDB Consultancy, Uppsala, Sweden ) solution was added to the AP chamber, and 1000 µL HBSS buffer was added to the BL chamber, and then the cells were cultured at 37 °C for 120 min. After incubation, a 200 µL BL chamber transport medium was collected in all-black 96-well plates for fluorescence intensity measurement and the related apparent permeability coefficient calculation. Three parallel plates were set for each group.

### 4.11. Data Analysis

The experiments were conducted three times, and the results are expressed as the mean ± SD. Analysis between groups was conducted using Student’s *t*-test or one-way analysis of variance followed by Bonferroni post hoc test (SPSS, USA). The *p* values < 0.05 were considered statistically significant in the control.

## 5. Conclusions

Our study shows that fucoidan can be applied to the Caco-2 model, which had no effect on macromolecular protein and drug absorption and can successfully shorten the modeling period to 5 days. This effect of fucoidan will provide a new theoretical basis for the application of fucoidan and may help to provide a more cost-effective method for studying the intestinal activity of substances.

## Figures and Tables

**Table 1:** The apparent permeability coefficient (Papp) (×10^−7^ cm/s) of fluorescein sodium after treatment with different fucoidan concentrations at different times.

Time (min)	Fucoidan (µg/mL)			
	Control (7d)	100 (5d)	50 (5d)	25 (5d)
30	4.30 ± 1.54	3.26 ± 0.18	4.16 ± 0.71	3.69 ± 0.96
60	6.31 ± 1.42	4.84 ± 0.42	5.92 ± 0.33	5.23 ± 0.76
120	6.81 ± 1.26	5.81 ± 0.02	6.63 ± 0.20	6.49 ± 0.83
180	6.90 ± 1.40	6.07 ± 0.23	6.59 ± 0.39	6.52 ± 0.44

Note: The regression equation of fluorescein sodium was Y = 98,782X + 15,085 (R2 = 0.9999, range 0.1–50 ng/mL). Significance test: a *t*-test was conducted.

**Table 2:** The transmittance (RT) of fluorescein sodium (%) at a different time and con-centration of Fucoidan.

Time (min)	Fucoidan (µg/mL)			
	Control (7d)	100 (5d)	50 (5d)	25 (5d)
30	0.07 ± 0.027	0.06 ± 0.003	0.06 ± 0.012	0.07 ± 0.017
60	0.22 ± 0.049	0.17 ± 0.014	0.18 ± 0.012	0.20 ± 0.026
120	0.47 ± 0.087	0.40 ± 0.002	0.45 ± 0.014	0.46 ± 0.058
180	0.72 ± 0.145	0.63 ± 0.034	0.68 ± 0.04	0.68 ± 0.045

Significance test: a *t*-test was conducted.

**Table 3:** Differences in the apparent permeability coefficient (Papp) of FITC-transferrin absorption (×10^−5^ cm/s).

Time (min)	Fucoidan (µg/mL)	
	Control (7d)	50 (5d)
120	4.45 ± 0.18	4.61 ± 0.05

Note: The regression equation of FITC-transferrin was Y = 559,348X+ 5050.3 (R² = 0.9991, range 0.001–2.5 μg /mL) Significance test: a *t*-test was conducted.

**Table 4:** Differences in the Papp of FITC-dextran absorption (×10^−5^ cm/s).

Time (min)	Fucoidan (µg/mL)	
	Control (7d)	50 (5d)
120	4.56 ± 0.28	4.46 ± 0.57

Note: The regression equation of FITC-dextran was Y = 28,575X + 11,675 (R² = 0.9993, range: 0.01–10 μg/mL) Significance test: a *t*-test was conducted.

## Data Availability

Data is contained within the article.

## Abbreviations

AKP: alkaline phosphatase; AP: apical; BL: basolateral; DMEM: Dulbecco’s modified eagle medium; EDTA: ethylenediamine tetraacetic acid; Fuc-PM-DMEM: Dulbecco’s modified eagle medium contain 0.4-µg/mL puromycin and fucoidan; P-GP: p-glycoprotein; PM: puromycin; PM-DMEM: Dulbecco’s modified eagle medium contain 0.4-µg/mL puromycin; SEM: Scanning electron microscopy; TEER: transepithelial electrical resistance; MTT: 3-(4,5-dimethylthiazol-2yl)-3,5-diphenytetrazolium bromide; Papp: apparent permeability coefficient; and RT: transmittance.
